# An artificial intelligence-based deep learning algorithm for the diagnosis of diabetic neuropathy using corneal confocal microscopy: a development and validation study

**DOI:** 10.1007/s00125-019-05023-4

**Published:** 2019-11-12

**Authors:** Bryan M. Williams, Davide Borroni, Rongjun Liu, Yitian Zhao, Jiong Zhang, Jonathan Lim, Baikai Ma, Vito Romano, Hong Qi, Maryam Ferdousi, Ioannis N. Petropoulos, Georgios Ponirakis, Stephen Kaye, Rayaz A. Malik, Uazman Alam, Yalin Zheng

**Affiliations:** 1 Department of Eye and Vision Science, University of Liverpool, William Henry Duncan Building, 6 West Derby Street, Liverpool, L7 8TX UK; 2 St Paul’s Eye Unit, Royal Liverpool University Hospital, Liverpool, UK; 3 Data Science Institute, Lancaster University, Lancaster, UK; 4 Department of Ophthalmology, Riga Stradins University, Riga, Latvia; 5 Department of Ophthalmology, Peking University Third Hospital, Beijing, China; 6 Cixi Institute of Biomedical Engineering, Ningbo Institute of Industrial Technology, Chinese Academy of Sciences, Ningbo, China; 7 Laboratory of Neuro Imaging, Institute for Neuroimaging and Informatics, Keck School of Medicine, University of Southern California, Los Angeles, CA USA; 8 Department of Endocrinology and Diabetes, University Hospital Aintree, Longmoor Lane, Liverpool, UK; 9 Weill Cornell Medicine – Qatar, Doha, Qatar; 10 Diabetes and Neuropathy Research, Department of Eye and Vision Sciences and Pain Research Institute, Institute of Ageing and Chronic Disease, University of Liverpool and Aintree University Hospital NHS Foundation Trust, William Henry Duncan Building, 6 West Derby Street, Liverpool, L7 8TX UK; 11 Department of Diabetes and Endocrinology, Royal Liverpool and Broadgreen University NHS Hospital Trust, Liverpool, UK; 12 Division of Endocrinology, Diabetes and Gastroenterology, University of Manchester, Manchester, UK

**Keywords:** Corneal confocal microscopy, Corneal nerve, Deep learning, Diabetic neuropathy, Image processing and analysis, Image segmentation, Ophthalmic imaging, Small nerve fibres

## Abstract

**Aims/hypothesis:**

Corneal confocal microscopy is a rapid non-invasive ophthalmic imaging technique that identifies peripheral and central neurodegenerative disease. Quantification of corneal sub-basal nerve plexus morphology, however, requires either time-consuming manual annotation or a less-sensitive automated image analysis approach. We aimed to develop and validate an artificial intelligence-based, deep learning algorithm for the quantification of nerve fibre properties relevant to the diagnosis of diabetic neuropathy and to compare it with a validated automated analysis program, ACCMetrics.

**Methods:**

Our deep learning algorithm, which employs a convolutional neural network with data augmentation, was developed for the automated quantification of the corneal sub-basal nerve plexus for the diagnosis of diabetic neuropathy. The algorithm was trained using a high-end graphics processor unit on 1698 corneal confocal microscopy images; for external validation, it was further tested on 2137 images. The algorithm was developed to identify total nerve fibre length, branch points, tail points, number and length of nerve segments, and fractal numbers. Sensitivity analyses were undertaken to determine the AUC for ACCMetrics and our algorithm for the diagnosis of diabetic neuropathy.

**Results:**

The intraclass correlation coefficients for our algorithm were superior to those for ACCMetrics for total corneal nerve fibre length (0.933 vs 0.825), mean length per segment (0.656 vs 0.325), number of branch points (0.891 vs 0.570), number of tail points (0.623 vs 0.257), number of nerve segments (0.878 vs 0.504) and fractals (0.927 vs 0.758). In addition, our proposed algorithm achieved an AUC of 0.83, specificity of 0.87 and sensitivity of 0.68 for the classification of participants without (*n* = 90) and with (*n* = 132) neuropathy (defined by the Toronto criteria).

**Conclusions/interpretation:**

These results demonstrated that our deep learning algorithm provides rapid and excellent localisation performance for the quantification of corneal nerve biomarkers. This model has potential for adoption into clinical screening programmes for diabetic neuropathy.

**Data availability:**

The publicly shared cornea nerve dataset (dataset 1) is available at http://bioimlab.dei.unipd.it/Corneal%20Nerve%20Tortuosity%20Data%20Set.htm and http://bioimlab.dei.unipd.it/Corneal%20Nerve%20Data%20Set.htm.

**Electronic supplementary material:**

The online version of this article (10.1007/s00125-019-05023-4) contains peer-reviewed but unedited supplementary material, which is available to authorised users.

## Introduction

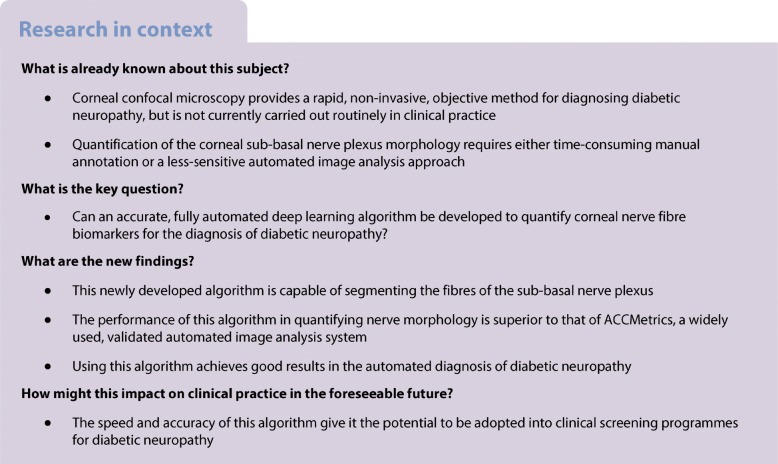



The prevalence of diabetic peripheral neuropathy (DPN) can be as high as 50% in an unselected population. Currently, screening for DPN most commonly relies on the 10 g monofilament test, which identifies individuals at risk of foot ulceration but is poor at identifying those with early neuropathy [1]. Screening methods such as clinical examination, questionnaires and vibration perception threshold do not provide direct quantification of small nerve fibres, which are the earliest site of injury. Skin biopsy enables direct visualisation of thinly myelinated and unmyelinated nerve fibres, which are the earliest affected in DPN, and can be used to diagnose small-fibre neuropathy (SFN) [2]. The assessment of intra-epidermal nerve fibres (IENFs) and IENF density is currently advocated in clinical practice in the USA [3] and recommended as an endpoint in clinical trials [4]. However, skin biopsy is invasive and requires specialised laboratory facilities for analysis. The cornea is the most densely innervated tissue of the human body, containing a network of unmyelinated axons (small nerve fibres) called the sub-basal nerve plexus (SBP).

**Fig. 1:**
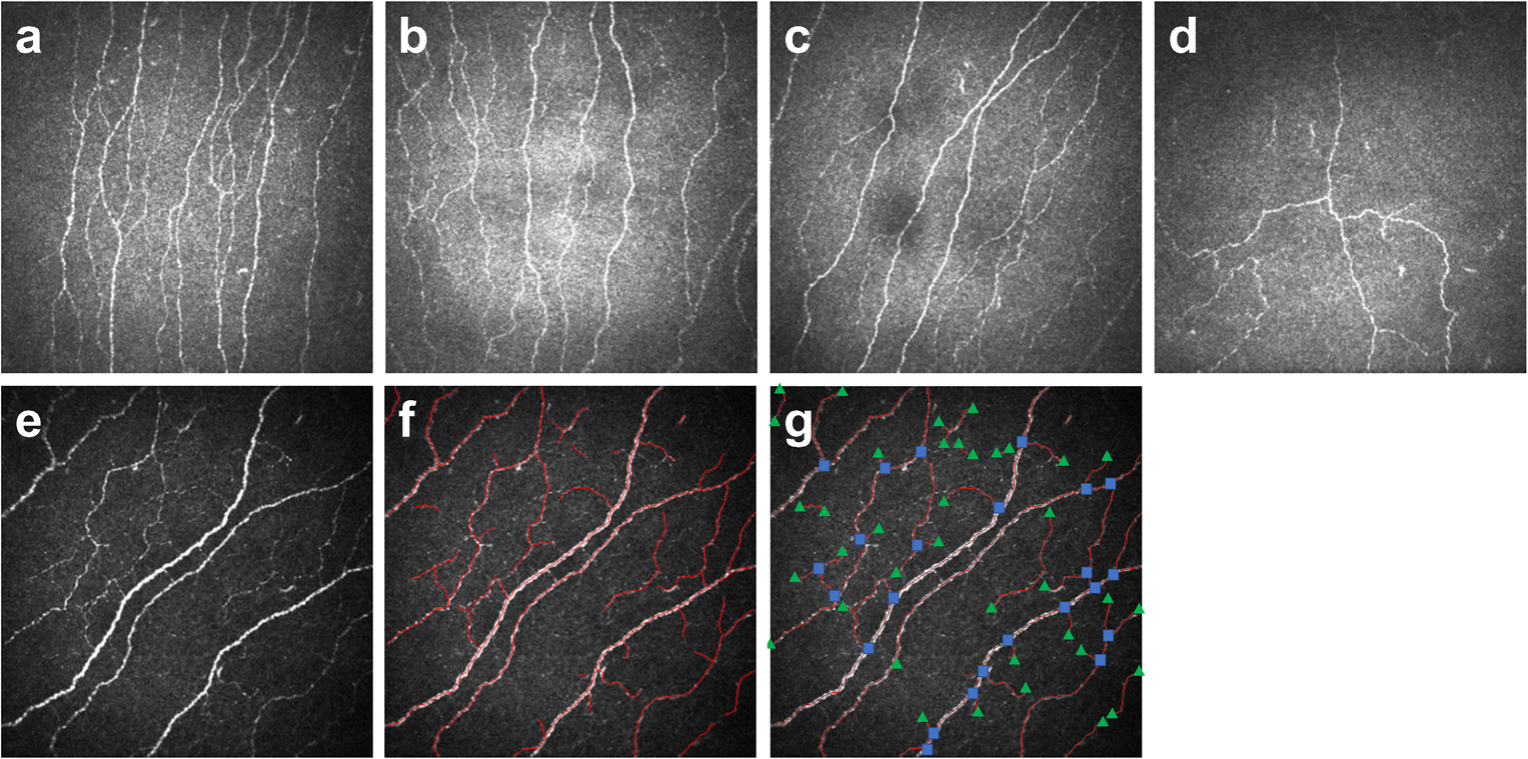
(**a**–**d**) Examples of CCM images from healthy individuals (**a**, **b**) and individuals with diabetic neuropathy (**c**, **d**). (**e**, **f**) An example image (**e**) with manual annotation (**f**) is shown. (**g**) Branch and terminal points (manually added) are shown, with green triangles denoting tail points and blue squares denoting branching points

Corneal confocal microscopy (CCM) has been used to image the SBP, which has been shown to be remarkably stable in healthy corneas over 3 years [5] but demonstrates early and progressive pathology in a range of peripheral and central neurodegenerative conditions [6–11]. Figure 1a,b and Fig. 1c,d show examples from participants without and with diabetic neuropathy, respectively. Previous studies have demonstrated analytical validation by showing that CCM reliably quantifies early axonal damage in DPN [12, 13] with high sensitivity and specificity [14, 15] and closely correlates to the loss of IENFs [15, 16]. CCM also predicts incident diabetic neuropathy [17] and can detect corneal nerve regeneration in people with DPN [18]. CCM may also detect early nerve fibre loss before IENF loss in skin biopsy [19]. In some individuals, corneal nerve fibre (CNF) loss may be the first evidence of subclinical DPN [19]; Brines et al [20] have shown that determination of CNF area and width distribution may improve the diagnostic and predictive ability of CCM.

To accurately quantify CNF morphology, nerves must be distinguished from background and other cell structures accurately. A major limitation for wider clinical utilisation is the need for manual image analyses, which is highly labour-intensive and requires considerable expertise to quantify nerve pathology [21]. The development of methods for the objective, reliable and rapid analysis of corneal nerves is vital if CCM is to be adopted for screening and large clinical trial programmes. Furthermore, to be used as a diagnostic tool, it is essential to extract the measurements automatically with high reliability [21]. Dabbah et al [22] presented a dual-model automated detection method for CNFs using CCM, showing excellent correlation with manual ground-truth analysis (*r* = 0.92). They further refined this method, using the dual-model property in a multi-scale framework to generate feature vectors from localised information at every pixel, and achieved an even stronger correlation with the ground-truth (*r* = 0.95) [21]. This study, however, used neural networks without convolution layers [21], which necessitates pre-processing and encourages overfitting.

Kim and Markoulli [23] developed a nerve segmentation technique to delineate corneal nerve morphology in CCM. This involved processes ranging from filtering methods (with rapid implementation but low-contrast and imprecise focus) to more complex support vector machine approaches (which rely on features defined by the user). Chen et al [24] presented a method based on feature engineering, achieving state-of-the-art results, although its reliance on hand-crafted features increases the complexity to the user and can introduce user-bias, returning suboptimal results [25].

Recently, approaches based on machine learning have achieved excellent performance in computer vision and medical image analysis tasks. Deep learning and, particularly, convolutional neural networks (CNNs; a class of deep neural networks) have emerged as a highly effective branch of machine learning for image classification [25]. This approach allows for ‘end-to-end’ classification results to be achieved without the need for specifying or designing features or setting example-specific parameters. CNN design follows vision processing in living organisms [26], with the connectivity pattern between neurons resembling visual cortex organisation. Based on training with pre-annotated data, CNNs combine the traditionally separate machine-learning tasks of feature designing, learning and image classification in a single model, relieving the traditional machine-learning burden of designing hand-crafted features. More recently, this has extended beyond image-wise classification to efficient pixel-wise classification, allowing image segmentation to be achieved (i.e. pixels may be classed as belonging or not belonging to an object of interest). There has been a significant increase recently in the development of deep learning algorithms (DLAs) with CNNs, an approach that achieves excellent performance in many computer vision applications and has clinical utility in healthcare [27]. Compared with manual detection, accurate automated detection of corneal nerves using CCM has many potential benefits, including objectivity, increased efficiency and reproducibility, allowing enhanced early disease diagnostics and improved patient outcomes. Artificial intelligence-based DLAs have the added advantage of continual learning and refinement alongside concurrent analysis.

The aim of this study was to develop and validate a DLA for corneal nerve segmentation in CCM images and to compare this with the widely used and validated automated image analysis software, ACCMetrics (Early Neuropathy Assessment [ENA] group, University of Manchester, Manchester, UK) [24].

## Methods

### Participants

All participants gave informed consent at the respective institutions and the studies were conducted in accordance with the Declaration of Helsinki. Relevant ethical and institutional approvals were gained prior to the imaging of all participants.

### Image datasets

In this study, 3835 confocal images of the corneal SBP were utilised from healthy volunteers and people with diabetes from Padova, Italy (*n* = 120), Beijing, China (*n* = 1578) and Manchester, UK (*n* = 2137). Figure 1e shows an example CCM image; Fig. 1f shows the manual annotation, with branching and tail points highlighted in Fig. 1g.

#### Dataset 1 (BioImLab, University of Padova, Italy)

One hundred and twenty images were obtained from Ruggeri’s BioImLab at the Department of Information Engineering, University of Padova, Italy. Of these, the first 30 images were from 30 volunteers (one image per person) who were either healthy or showed different pathologies (diabetes, pseudoexfoliation syndrome, keratoconus) [28]. The images were captured in TIFF format at 384×384 pixels with a Heidelberg Retina Tomograph II using the Rostock Corneal Module (RCM; HRTII32-RCM) confocal laser microscope (Heidelberg Engineering, Heidelberg, Germany). The remaining 90 images were of the corneal sub-basal epithelium from individuals displaying normal or abnormal pathologies, with one image per person [29], using a ConfoScan 4 CCM at ×40 magnification (Nidek Technologies, Padova, Italy). An area of 460×350 μm was captured at 768×576 pixels and stored in monochrome JPG compressed format.

#### Dataset 2 (Peking University Third Hospital, Beijing, China)

One thousand, five hundred and seventy-eight images (384×384 pixels in TIFF format) were acquired from healthy volunteers (*n* = 90) and from individuals with corneal abnormalities (*n* = 105, including 52 participants with diabetes) using the Heidelberg Retina Tomograph 3/RCM (Heidelberg Engineering, Heidelberg, Germany). Six images per eye were obtained where possible from the corneal apex using the same methodology developed and utilised by the ENA group (University of Manchester, Manchester, UK).

#### Dataset 3 (ENA Group, University of Manchester, UK)

Two thousand, one hundred and thirty-seven images were analysed from healthy volunteers and participants with diabetes (*n* = 444). All CCM images were obtained using the standard, internationally accepted protocol developed by the ENA group [13]. The images (400×400 μm [384×384 pixels]) were captured using the RCM set at +12 objective lens. The images were exported in BMP format, which is compatible with the image analysis software. Images were from the following cohorts: group 1, healthy volunteers (*n* = 90); group 2, participants with impaired glucose tolerance (*n* = 53, including 26 with definite neuropathy); group 3, participants with type 1 diabetes with definite neuropathy (*n* = 37); group 4, participants with type 1 diabetes without neuropathy (*n* = 53); group 5, participants with type 2 diabetes without (*n* = 101) and with definite neuropathy (*n* = 49); group 6, participants with type 2 diabetes and with mild neuropathy (*n* = 41) and definite neuropathy (*n* = 20). Definite neuropathy was defined as the presence of an abnormality in nerve conduction studies as per age-related reference range and symptom(s) or sign(s) of neuropathy as defined by the Toronto Consensus statement by the American Diabetes Association on DPN [30]. Across the groups, a total of 132 participants had definite neuropathy. Note that the depth of images were only marginally different for each participant and depended on corneal thickness. However, the SBP occurs at a depth of ~50 μm in most people, regardless of the presence or absence of diabetes [31].

### Image annotation

To obtain a ground-truth for each image in the BioImLab and Beijing datasets, the corneal nerves in each image were manually traced by a clinical ophthalmologist (DB) using an in-house program written in Matlab (Mathworks R2017, Natick, MA, USA). Our previous work has demonstrated the validity of manual annotations in terms of intra- and inter-observer agreements [32]. Dataset 3 was not annotated and was only used for clinical testing using the deep learning segmentations.

### Automatic segmentation of the corneal nerves in CCM images

The preparation of our combined datasets for use in a training and testing approach is presented in this section, and we define our automated segmentation method. This is then built on with ensemble learning and random sampling. Finally, clinically relevant variables were extracted and compared with those obtained using existing state-of-the-art ACCMetrics.

#### Dataset preparation for training/testing approach

The BioImLab and Beijing datasets included 1698 images in total and were used for the development of the model: 1494 (~90%) images from the Beijing dataset were used for training, while 84 images from Beijing and all the BioImLab dataset were used for testing. Each image in these datasets was used for either training or testing to avoid overfitting. Dataset 3 (ENA image dataset) was only used for clinical testing and validation but not to train the model. The images for training and testing were selected using a random permutation at the individual level determined using a Mersenne Twister method [33]. Note that splitting took place on the image (rather than individual) level in order to avoid potential bias.

All the images were standardised to have a pixel size of 1.04 μm (384×384 pixels) by bilinear interpolation. To increase the dataset size, it was augmented by extracting patches of size 128×128 pixels with an overlap of 32 pixels, creating 81 patches per image. The selection of patches used for training/testing was done at the image level to avoid testing patches from images whose data had been used for training.

#### Image segmentation using deep learning

**Fig. 2:**
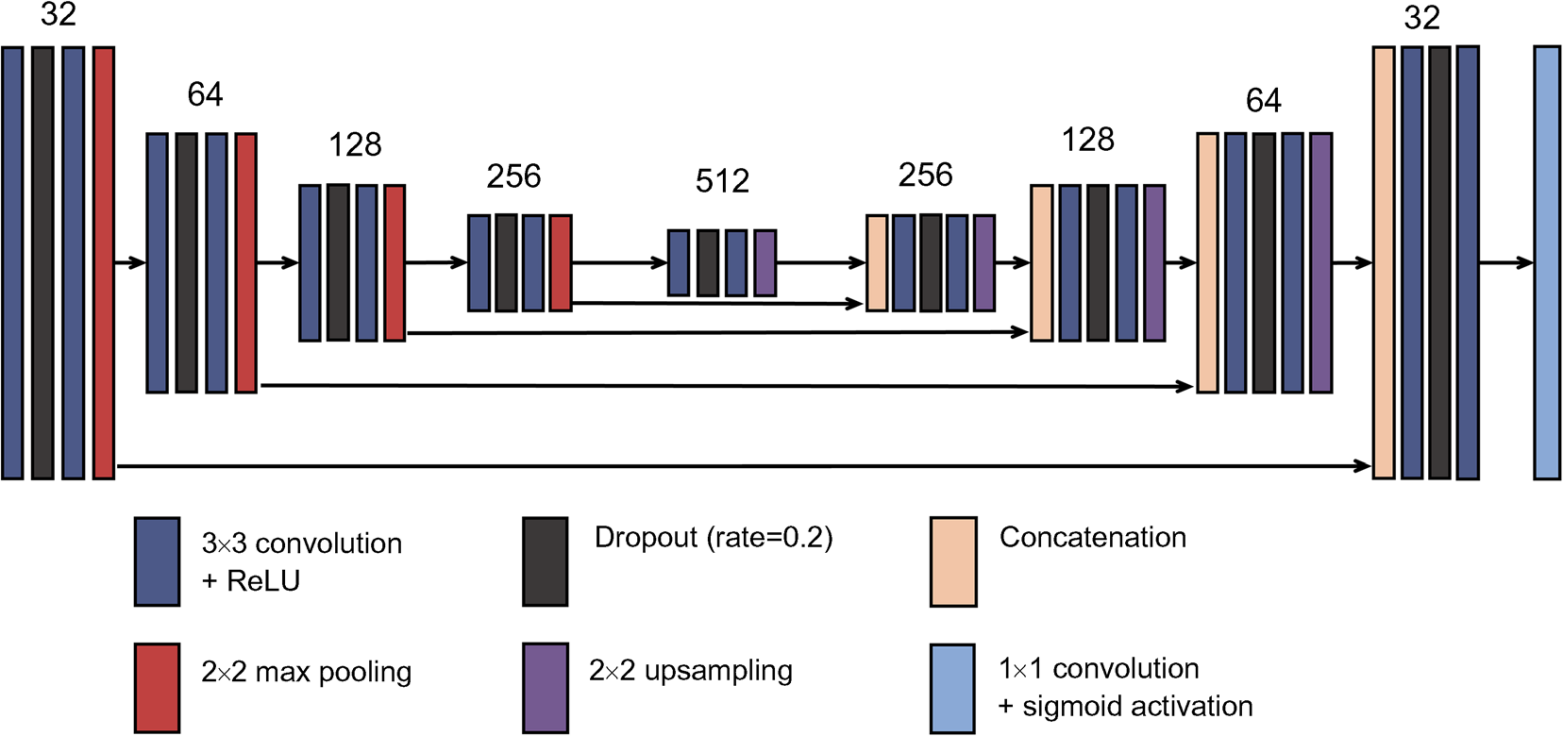
Diagram of the proposed U-Net architecture. Each dark blue rectangular block corresponds to a multi-channel features map passing through 3×3 convolution followed by rectified linear unit (ReLU) operations. Dark grey blocks denote dropout operation with a rate of 0.2. Red and purple blocks denote 2×2 max pooling and upsampling, respectively. Light brown blocks denote the concatenation of feature maps. The light blue block denotes a 1×1 operation followed by sigmoid activation. The number of channels is indicated at the top of each column

Corneal nerves were segmented adopting U-Net CNN architecture [34]. Unlike conventional CNNs, which aim to assign one classification (or more) to an image, this type of architecture aims to achieve full-image segmentation by determining a pixel-wise segmentation map. Figure 2 illustrates the architecture of our proposed U-Net model. It can be visualised as a U-shape, the left side being an encoding path and the right side a decoding path. At the end of the architecture, a sigmoid activation function is employed to create a segmentation map. A key feature of U-Net is direct connectivity between the encoding and decoding layers, allowing extracted feature re-use and strengthening feature propagation. The Dice similarity coefficient (DSC) was used as a cost function (i.e. to measure error during training).

The models were developed with Python 3.5.2 (https://www.python.org/), Tensorflow 1.0.0 (https://www.tensorflow.org/; Google, Mountain View, CA, USA) and Keras 1.2.1 (https://keras.io/) and trained for 200 iterations using an NVIDIA K40 GPU (NVIDIA, Santa Clara, CA, USA). Following the training step, the trained model weights were used to obtain the segmentation maps of each previously unseen testing patch.

#### Ensemble classification

To improve the accuracy of the model by using multiple copies, an ensemble of five U-Net networks were trained on the same training data using a random-sample-with-replacement approach. The final prediction was computed by a majority vote over the predictions of the ensemble network. Henceforward in this manuscript, training with our preliminary model, a single U-Net model, is referred to as the ‘Liverpool Convolutional Neural Network’ (LCNN), while training with our refined ensemble deep learning approach is referred to as the ‘Liverpool Deep Learning Algorithm’ (LDLA).

#### Image reconstruction and variable extraction

The trained models were able to produce segmentations on a patch basis. The segmentation of a whole CCM image was obtained by combining the segmentations of all its patches using majority voting on the overlap regions. From the image-level segmentation result, further analysis was carried out to derive the clinically relevant variables including the corneal nerve length, branch points, tail points and fractal number [35].

#### Evaluation

Our LCNN and LDLA models, together with the state-of-the-art ACCMetrics model (ACCM) [24], were compared with the manual annotation. The performance of the algorithm was measured using the Bland–Altman approach. Agreement between the automatic segmentations and manual annotations were assessed using the intraclass coefficient (ICC). For the clinical evaluations, ANOVA with Tukey post hoc analysis was performed for comparison between different groups of participants. The AUC was calculated to compare the detection performance of different models. SPSS for Windows, version 22.0 (IBM-SPSS, Chicago, IL, USA) was used for the statistical analysis, with a *p* value of < 0.05 deemed statistically significant.

## Results

**Fig. 3:**
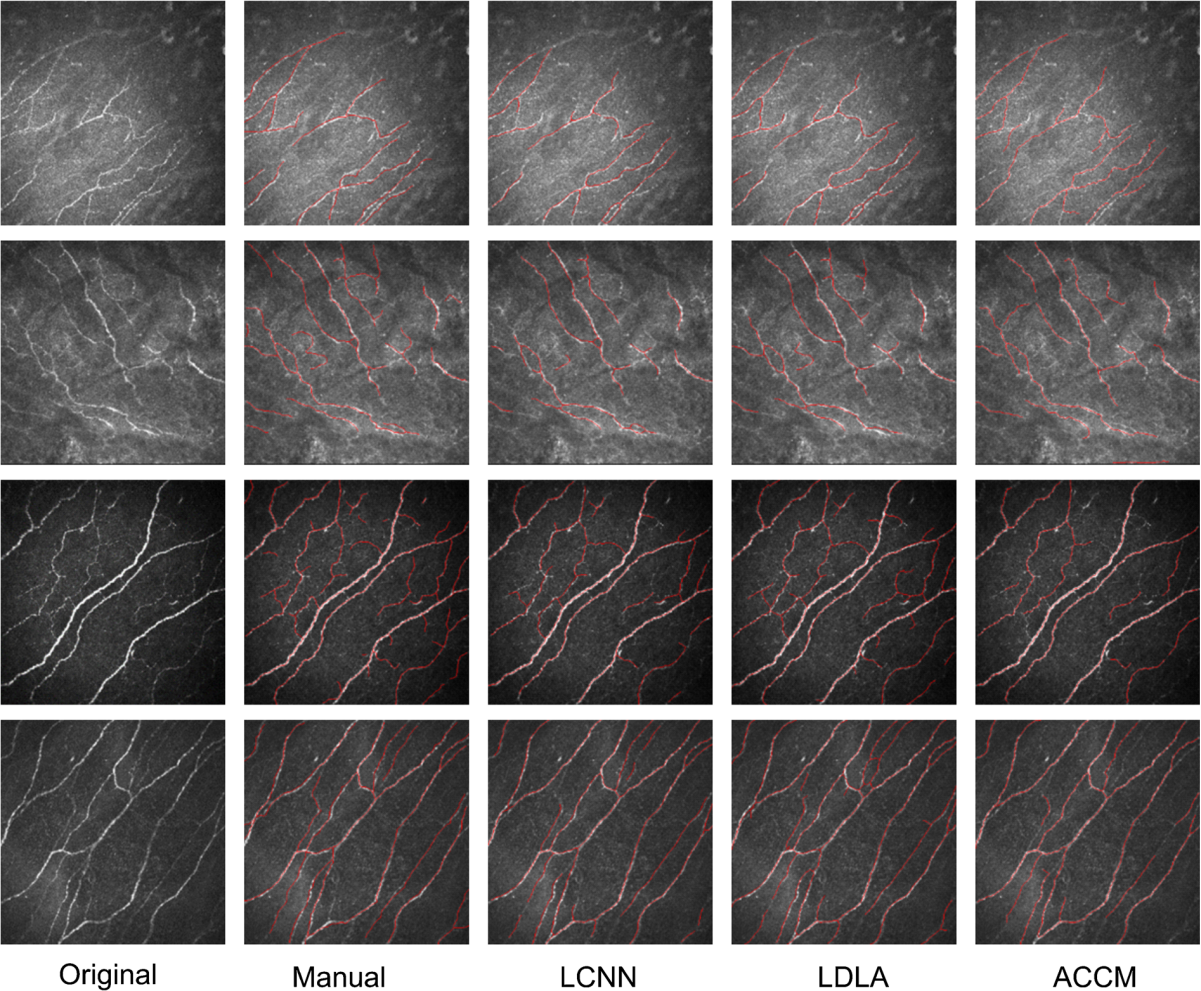
Four examples of segmentation of corneal nerves. Columns appear in the following order: the original images; manual annotations; and segmentation results of the LCNN model, LDLA and ACCM, respectively. Red lines denote the centre lines of the segmented nerves

Figure 3 shows four example testing images along with their ‘ground-truth’ manual annotations and segmentation results obtained by LCNN, LDLA and ACCM. LCNN and LDLA produced results that were more faithful to the manual annotations than did the ACCM, particularly in example 4 where the ACCM failed to detect nerves at the top middle and right bottom of the image. Overall, the segmentation performance was consistent and there was no obvious failed case. For illustration, electronic supplementary material [ESM] Fig. 1 shows all the first 30 images of dataset 1, ESM Fig. 2 shows the 12 randomly chosen images from dataset 2, and ESM Fig. 3 shows 12 randomly chosen images from dataset 3.

**Fig. 4:**
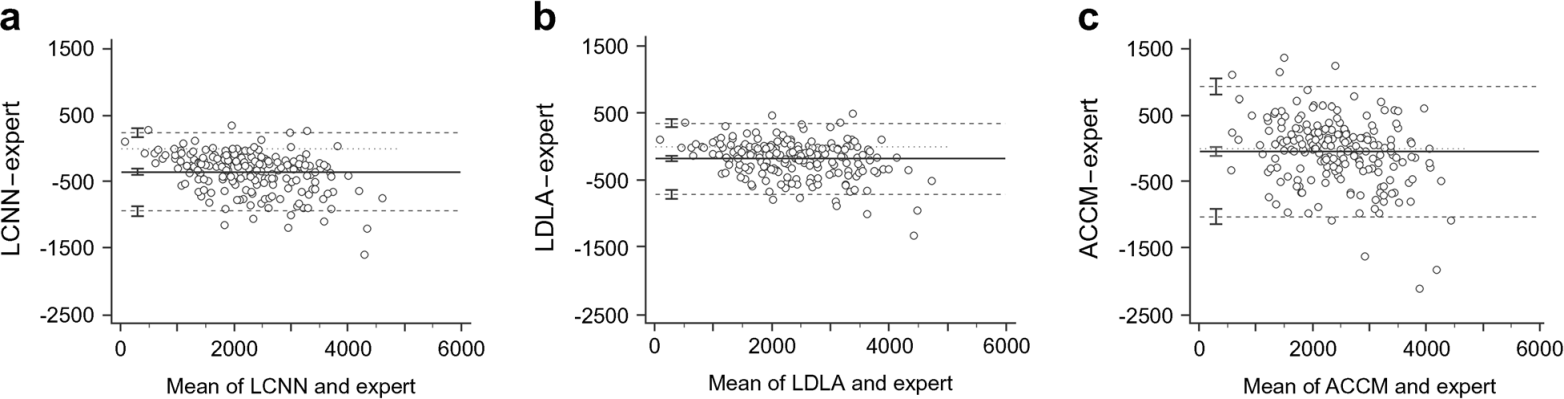
Bland–Altman plots showing the difference in determination of the total CNF length (μm) between the LCNN (**a**), LDLA (**b**) and ACCM (**c**) methods and manual annotations by an expert with clinical expertise. The limits of agreement are defined as the mean difference ± 1.96 SD of the differences. Error bars represent the 95% CI for the mean and both the upper and lower limits of agreement

Analysis of datasets 1 and 2 shows that the mean total CNF length from the manual ‘ground-truth’ annotation was highest (2441.4±919.5 μm) compared with the three automated approaches (LCNN 2089.4±804.6 μm; LDLA 2260.3±835.3 μm; ACCM 2394.1±768.1 μm). Total CNF length was greater in the ACCM and was closer to the total length from the manual annotation than were the LCNN or LDLA results. However, ICC analysis (Table 1) demonstrated that the LCNN and LDLA both produced results more consistent with the manual annotations when compared with the ACCM. Furthermore, our two methods performed consistently better than the ACCM in terms of correct segment length, number of branching points and fractal numbers. Bland–Altman analysis (Fig. 4) further confirmed that the limits of agreement of the ACCM were greater than those of both the LCNN and LDLA, implying greater variability despite the mean total corneal lengths in the ACCM. In other words, although the results of the ACCM were closer to the manual annotation in this case, the variation due to over- and under-segmentation was much larger than either the LCNN or the LDLA; the ACCM may therefore have produced heterogeneous results.

**Table 1:** Absolute agreement measured by ICC

Method	Total length	Mean length per segment	No. of branch points	No. of tail points	No. of nerve segments	Fractal
LCNN	0.867	0.596	0.809	0.647	0.844	0.887
LDLA	0.933	0.656	0.891	0.623	0.878	0.927
ACCM	0.825	0.352	0.570	0.257	0.504	0.758

Based on the 95% CI of the ICC estimate, a value of <0.5, 0.5–0.75, 0.75–0.9 and >0.90 is indicative of poor, moderate, good and excellent reliability, respectively [36].

Table 2 shows the comparisons of the root mean square error and SD of the derived measures *v*_*i*_ over each image *i*, against the manual annotations, in terms of number of branching points, number of terminal points, number of segments, total nerve fibre length, mean nerve fibre length, SD of nerve fibre length, and fractal number for each of the methods *M* using:

$$ \mathrm{RMSE}=\sqrt{\frac{1}{n}\sum \limits_i{V}_{i,M}^2},\kern0.5em \mathrm{SD}=\sqrt{\frac{1}{n-1}\sum \limits_i{\left({V}_{i,M}-\overline{V_M}\right)}^2},\kern0.5em \overline{V}=\sum \limits_i{V}_{i,M},\kern0.5em V={v}_{i,M}-{v}_{i, MA} $$

**Table 2:** RMSE and SD of the error of each of the methods for different measures

Variable	LCNN	LDLA	ACCM
No. of branching points			
RMSE	5.1326	4.1603	7.3667
SD	4.3934	4.1652	6.9110
No. of terminal points			
RMSE	8.7127	8.6766	13.0271
SD	8.0985	8.3479	11.6407
No. of segments			
RMSE	8.9521	8.3752	15.0708
SD	8.4248	8.3929	15.0296
Total fibre length			
RMSE	463.4712	326.0016	501.6230
SD	302.2312	271.7448	500.6295
Mean fibre length			
RMSE	40.0348	37.6684	51.1406
SD	37.9770	34.3762	50.6172
Standard deviation of fibre length			
RMSE	27.6372	24.0555	33.4168
SD	26.8020	22.6133	32.4616
Fractal number			
RMSE	0.0403	0.0307	0.0518
SD	0.0278	0.0273	0.0519

Lower values indicate closer agreement with the manual annotation

As shown in Table 2, LDLA had lower values for every measure, indicating closer agreement with the ground-truth annotation. For each measure, the LCNN had the second-lowest root mean squared error (RMSE) and the ACCM had the highest, indicating weaker agreement. The LDLA had the lowest SD for all measures except the number of terminal points, indicating more consistent agreement with the ground-truth over the set of images; the ACCM had the highest SD for all measures. From this, it can be concluded that both the LCNN and the LDLA outperform the ACCM and that the LDLA clearly has the best performance.

Given the convincing performance of the LDLA, which outperforms both the LCNN and the ACCM in each metric, it was applied to the third dataset and the results were used for clinical evaluation.

### Clinical testing and validation based on ENA image dataset

**Fig. 5:**
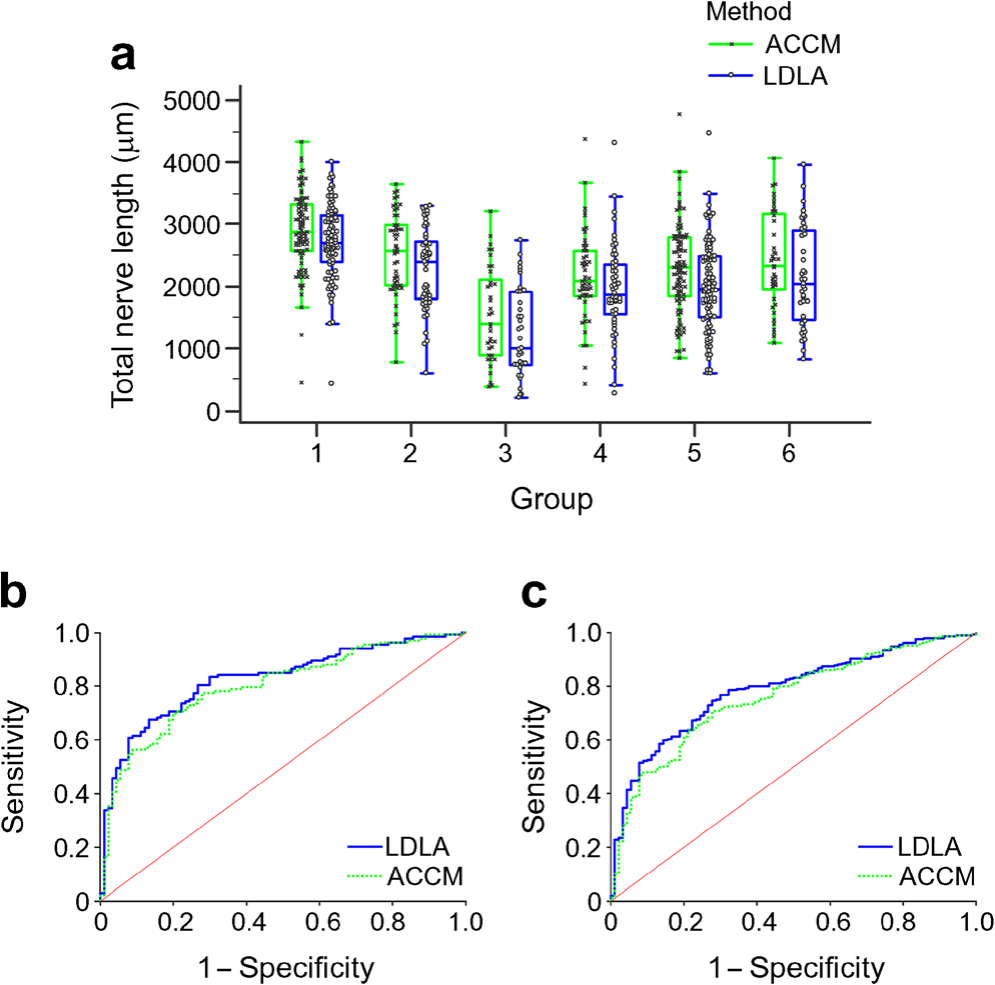
Analysis of total CNF length for the participants in dataset 3. (**a**) Box plot in combination with dot plot of the total CNF length in the six groups determined using our LDLA and the ACCM. The line within each box represents the median, and the top and bottom of the box represent the 75th and 25th percentiles, respectively. The whiskers indicate the maximum and minimum values excluding outliers. Group 1, healthy; group 2, impaired glucose tolerance; group 3, type 1 diabetes with definite neuropathy; group 4, type 1 diabetes without neuropathy; group 5, type 2 diabetes without and with definite neuropathy; group 6, type 2 diabetes with mild neuropathy and definite neuropathy. (**b**) ROC curves of classification of participants without and with diabetic neuropathy, comparing the LDLA and the ACCM. (**c**) ROC curves of classification of participants with and without diabetes, comparing the LDLA and the ACCM

ANOVA analysis demonstrated that differences in the total CNF length between the six groups of participants (in dataset 3) are in keeping with their neuropathy phenotype (Table 3, Fig. 5). A Tukey post hoc analysis was performed, and demonstrated that the CNF length in healthy volunteers was higher than in all the other groups (*p* < 0.01) while the total CNF length in people with type 1 diabetes and neuropathy (group 3) was lower than in all other groups (*p* < 0.001). The ACCM consistently yielded higher total CNF length than the LDLA.

**Table 3:** Total CNF length for dataset 3 utilising the LDLA

Group^a^	No. of participants	Mean CNF length (μm)	SD
1	90	2695.2	606.8
2	53	2245.2	648.6
3	37	1229.0	710.4
4	53	1917.1	732.2
5	108	2000.4	710.0
6	41	2131.7	803.7
Total	382	2125.9	800.6

^a^Group 1, healthy; group 2, impaired glucose tolerance; group 3, type 1 diabetes with definite neuropathy; group 4, type 1 diabetes without neuropathy; group 5, type 2 diabetes without and with definite neuropathy; group 6, type 2 diabetes with mild neuropathy and definite neuropathy

AUC analysis was undertaken to compare the LDLA and ACCM results (Fig. 5b,c). First, total corneal nerve length alone was used to classify individuals without and with and neuropathy. There was a total of 132 individuals with neuropathy (from groups 2, 3, 5 and 6) and 90 without (group 1). The resulting receiver operating characteristic (ROC) curve in Fig. 5b shows that the AUC is 0.826 for the LDLA and 0.801 for the ACCM, respectively. To determine the sensitivity and specificity of the model, optimal cut points were determined by the commonly used Youden index [37] (i.e. the sum of sensitivity and specificity minus 1). In a perfect test, Youden’s index is equal to 1. For the LDLA, the optimal cut determined a specificity of 0.867 and sensitivity of 0.677, while the ACCM achieved a specificity of 0.800 and sensitivity of 0.699. The LDLA showed better prediction performance than the ACCM when utilising CNF length. Similarly, Fig. 5c shows that the LDLA had better prediction performance in classifying healthy volunteers (*n* = 90) and all participants with diabetes (*n* = 301 from groups 3, 4, 5 and 6) than the ACCM when utilising CNF length; the AUC was 0.806 for the LDLA and 0.780 for the ACCM. The optimal cut points of the LDLA were specificity 0.7222 and sensitivity 0.784, while for the ACCM they were specificity 0.7222 and sensitivity 0.745.

## Discussion

In this study, an artificial intelligence-based DLA has been developed for the analysis and quantification of corneal nerves in CCM images. To our knowledge, this is the first DLA for the analysis of corneal nerve morphology and pathology. This study validates our DLA and demonstrates its superior performance compared with ACCMetrics, the existing state-of-the-art system. In particular, there are more consistent results, as demonstrated by a superior intraclass correlation for a number of metrics including total CNF length. In addition to the total CNF length, this DLA is also capable of producing the number of branching and tail points, fractal numbers, tortuosity and segment length. As such, these quantitative variables may provide additional utility to diagnose diabetic neuropathy and neuropathic severity.

A fractal is a visual product of a non-linear system characterised by its complexity and by the quality of self-similarity or scale invariance. Fractal analysis of the corneal SBP has been proposed by several authors [38, 39]. We believe that the additional utility of fractal dimensions provide an additional means of differentiating individuals with early or subclinical DPN. CNF length is a robust measure of DPN and SFN. A large multicentre pooled concurrent diagnostic validity study revealed that CNF length was the optimal CCM variable [40]. CNF length has also been shown to be a measure of early small-fibre regeneration [41]. From published data, CNF length and density are the most robust measures of DPN. Our data confirms the validity of CNF length. However, we feel other metrics are also of importance and require further scientific interrogation in a real-world clinically oriented study.

In this study, the quantification of images demonstrates a reduction in total CNF length in individuals with diabetic neuropathy compared with healthy volunteers. This study is in keeping with other data on the utility of CNF length as a valid biomarker of diabetic neuropathy [11, 12, 15, 18]. The sensitivity and specificity of our DLA for gold-standard DPN diagnosis with CCM (using the Toronto criteria) is far superior to currently used clinical methods such as the 10 g monofilament and 128 Hz tuning fork [42] (with rudimentary clinical assessments), thus providing a strong rationale for its use in clinical screening/practice.

This study extends our preliminary work on 584 CCM images where the initial DLA demonstrated good localisation performance for the detection of corneal nerves [43]. Our preliminary model was refined to produce the ensemble (LDLA) model, now validated in large image datasets to diagnose diabetic neuropathy using CCM.

The strength of deep learning is echoed by Oakley et al [44] who used corneal nerve segmentation in CCM images from macaques. Deep-learning-based approaches make the segmentation task relatively easier for the end user compared with the conventional approaches employing various filters and graphs [23]. In particular, compared with conventional machine-learning methods, such as support vector machines (SVM), deep learning reduces the need and additional complexity of feature selection and extraction, allowing the computer to learn features alongside the segmentation. The training of deep learning approaches is computationally expensive (e.g. it takes approximately 30 min per epoch to train a single U-Net model). The advantage is that, once the model is trained, the segmentation is very fast, taking milliseconds to segment CCM images.

In recent years, CNNs and DLAs have been added to algorithms used to screen for diabetic retinopathy. DLAs promise to leverage the large number of images for physician-interpreted screening and learn from raw pixels. The high variance and low bias of these models will allow DLAs to diagnose diabetic neuropathy using CCM images without the pre-processing requirements and more-likely overfitting of earlier approaches [25]. This automated DLA for the detection of diabetic neuropathy offers a number of advantages including consistency of interpretation, high sensitivity and specificity, and near instantaneous reporting of results. In this study, good sensitivity and adequate specificities were achieved using our DLA.

This is the largest study, to date, of the development and validation of corneal nerve segmentation and supersedes the numbers in the study by Chen et al [24], who used 1088 images from 176 individuals, with 200 images for training and 888 for testing. Our study used a robust dataset; however, further development of this DLA requires use of a developmental set of images with large numbers (tens of thousands) of normal and abnormal pathologies. An area of further research is that of interrupted CNF segments, which have often proved challenging in the CNF segmentation results obtained using earlier methods [24]. This problem is mainly caused by non-uniform illumination and contrast variations of CNF in images. Since quantitative biomarkers like CNF length and density are important measures for computer-aided diagnosis, missing CNF segments may theoretically reduce the diagnostic reliability of any automated system. In our previous work, the automatic gap reconnection method proposed by Zhang et al [45] was employed to bridge the interrupted nerve fibre structures. The gap-filling task is achieved by enforcing line propagation using the stochastic contour completion process with iterative group convolutions. Geometric connectivity of local CNF structures can be easily recovered based on their contextual information [45]. However, this connection step was not included in this model as there was only a modest improvement in the quantification of CNF length despite extra computation time of about 1 min per image. This is an area for future development of the DLA. It will also be important to investigate the potential for bias to be introduced by factors such as camera type. The major advantage of this DLA over standard automated techniques is the continual learning and refinement of the algorithm.

Given that 420 million people worldwide have been diagnosed with diabetes mellitus [46] and that the prevalence of diabetic neuropathy is ~50% [47], there is a need for valid quantitative population screening for diabetic neuropathy to prevent or limit sequelae such as foot ulcers and amputations. Skin biopsy with quantification of IENFs has been considered the ‘reference standard’ test for the diagnosis of SFN [48]. It is an invasive test, needing specialist diagnostic facilities and repeated tests at the same site, which is not always feasible. CCM is a rapid non-invasive ophthalmic imaging modality, which quantifies early axonal damage in diabetic neuropathy with high sensitivity and specificity [12–16, 49]. CCM also predicts incident neuropathy [17] and accurately detects CNF regeneration [18, 50]. The utility of CCM in diagnosing and monitoring the progression of diabetic neuropathy has been extensively evaluated [11, 12, 15, 16, 18, 38].

Further studies are required to determine the feasibility of applying this algorithm in the clinical setting and to compare outcomes with those obtained from currently used diabetic neuropathy screening methods typically having low sensitivity for detection except in advanced neuropathy. There is also a need to compare the diagnostic ability of this DLA with tests of small-fibre dysfunction (thermal thresholds/sudomotor/autonomic) and IENF density in skin biopsy in DPN and other peripheral neuropathies. The next key step is also to utilise the DLA alongside clinical neuropathy screening in a multicentre primary care study.

### Conclusion

Automated detection and screening offer a unique opportunity to detect early neuropathy and prevent the sequelae of advanced diabetic neuropathy. Our results demonstrate that this artificial intelligence-based DLA provides excellent localisation performance for the quantification of corneal nerve variables and therefore has the potential to be adopted for screening and assessment of diabetic neuropathy.

#### Contribution statement

BW, YLZ, YTZ and JZ worked on the proposed model and conducted experimental testing. RL, BM and HQ worked on the acquisition of dataset 2. JL, MF, UA, IP, GP and RM worked on ACCMetrics and the collection and compilation of dataset 3. DB, VR and SBK worked on the annotations of dataset 3. All authors were involved in discussions regarding the work, the writing and revisions of the manuscript and approved the submitted version. YLZ and UA are joint guarantors of this manuscript.

## Electronic supplementary material

ESM figures (PDF 967 kb)

## Data Availability

The publicly shared cornea nerve dataset (dataset 1) is available at http://bioimlab.dei.unipd.it/Corneal%20Nerve%20Tortuosity%20Data%20Set.htm and http://bioimlab.dei.unipd.it/Corneal%20Nerve%20Data%20Set.htm. The remaining data are publicly available upon request with restrictions in accordance with ethics approvals. Data access request to the remaining data should be made to YLZ (yalin.zheng@liverpool.ac.uk) and UA (uazman.alam@liverpool.ac.uk) respectively.

## Abbreviations

ACCM: ACCMetrics model
CCM: Corneal confocal microscopy
CNF: Corneal nerve fibre
CNN: Convolutional neural network
DLA: Deep learning algorithm
DPN: Diabetic peripheral neuropathy
DSC: Dice similarity coefficient
ENA: Early neuropathy assessment
ICC: Intraclass coefficient
IENF: Intra-epidermal nerve fibre
LCNN: Liverpool Convolutional Neural Network
LDLA: Liverpool Deep Learning Algorithm
RCM: Rostock Corneal Module
RMSE: Root mean squared error
ROC: Receiver operating characteristic
SBP: Sub-basal nerve plexus
SFN: Small-fibre neuropathy
