# Supplementary material for: An artificial intelligence-based deep learning algorithm for the diagnosis of diabetic neuropathy using corneal confocal microscopy: a development and validation study
Source: Diabetologia. 2019 Nov 12;63(2):419–30. doi: 10.1007/s00125-019-05023-4 (PMC6946763; doi:10.1007/s00125-019-05023-4)
Supplement: Supplementary file 1 — (PDF 967 kb) [file 125_2019_5023_MOESM1_ESM.pdf]

In this supplementary material, we show further examples of our LDLA on all 30 images forming the first part of Dataset 1 from BioImLab [34] in ESM Figs. 1a and 1b, on 10 randomly selected images from Dataset 2 from Beijing in ESM Fig. 2, and on 10 randomly selected images from Dataset 3 in ESM Fig. 3.

ESM Fig. 1a

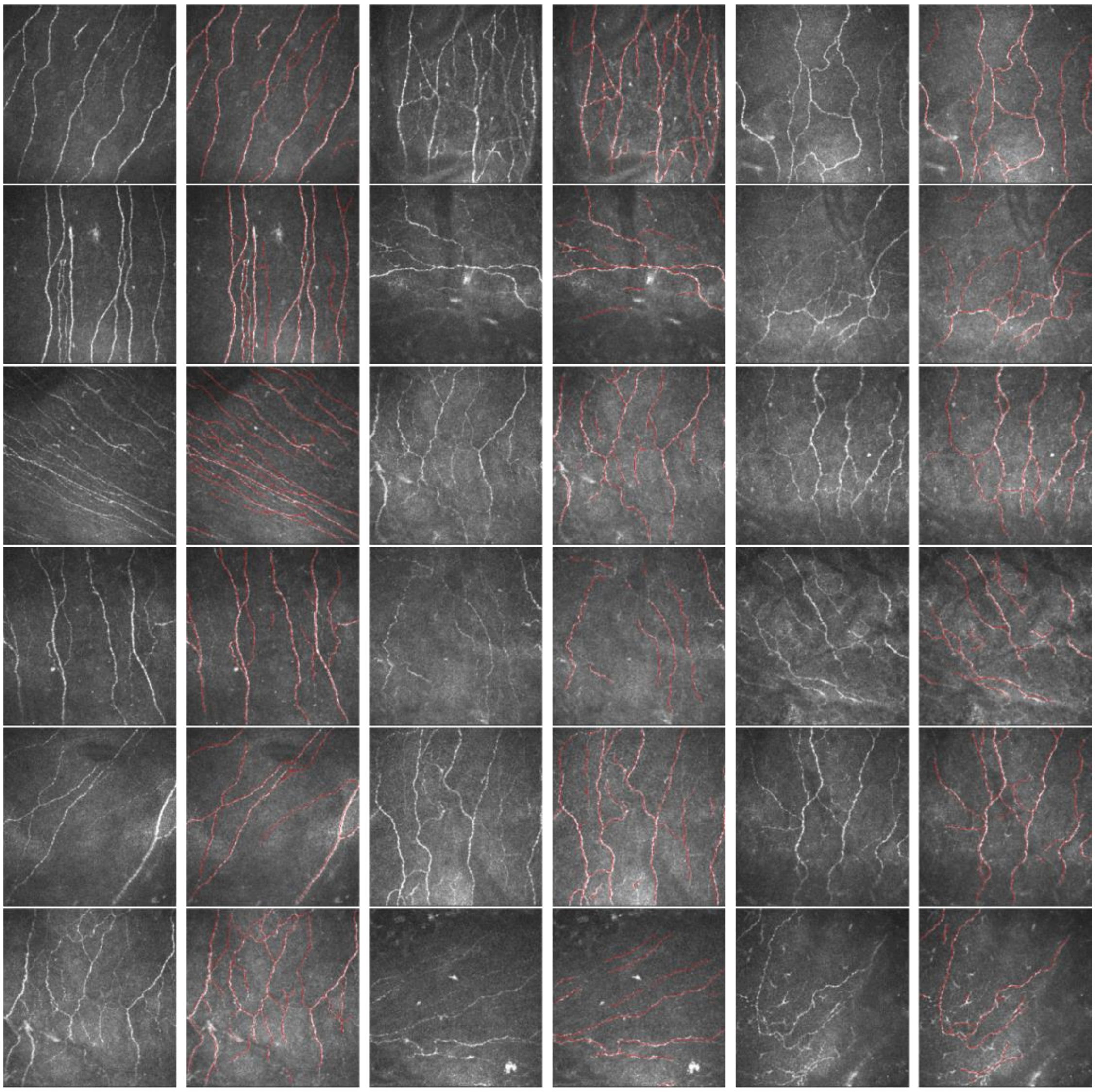

ESM Fig. 1a: Original and segmentation results from our LDLA overlaid (red) on 18 of the 30 images forming the first part of Dataset 1 from BioImLab [34].

ESM Fig. 1b

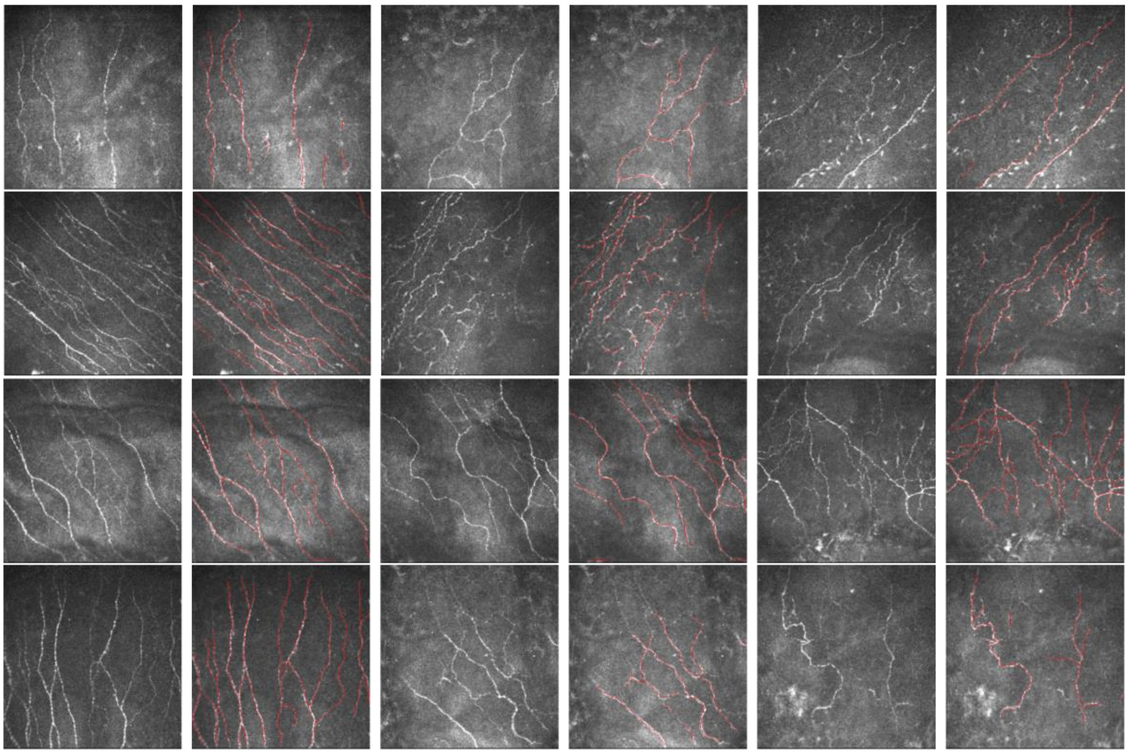

ESM Fig. 1b: Original and segmentation results from our LDLA overlaid (red) on 12 of the 30 images forming the first part of Dataset 1 from BioImLab [34].

ESM Fig. 2

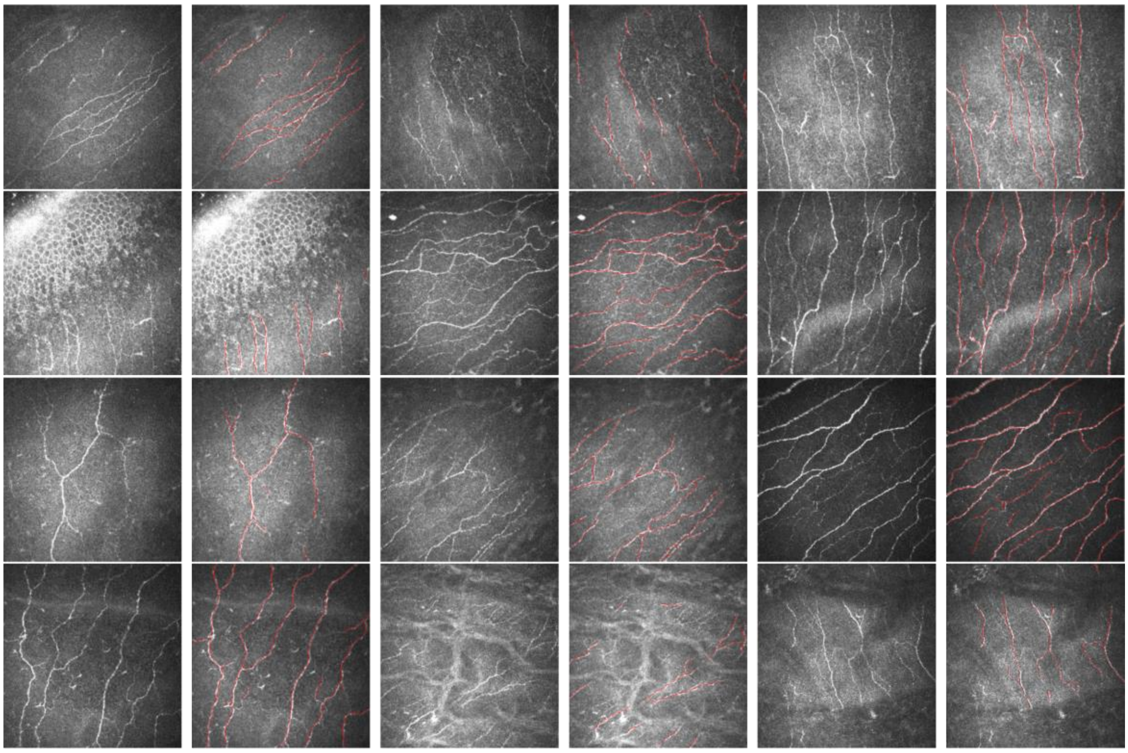

Figure SM2: Original and segmentation results from our LDLA overlaid (red) on 10 randomly selected images from Dataset 2 from Beijing.

ESM Fig. 3

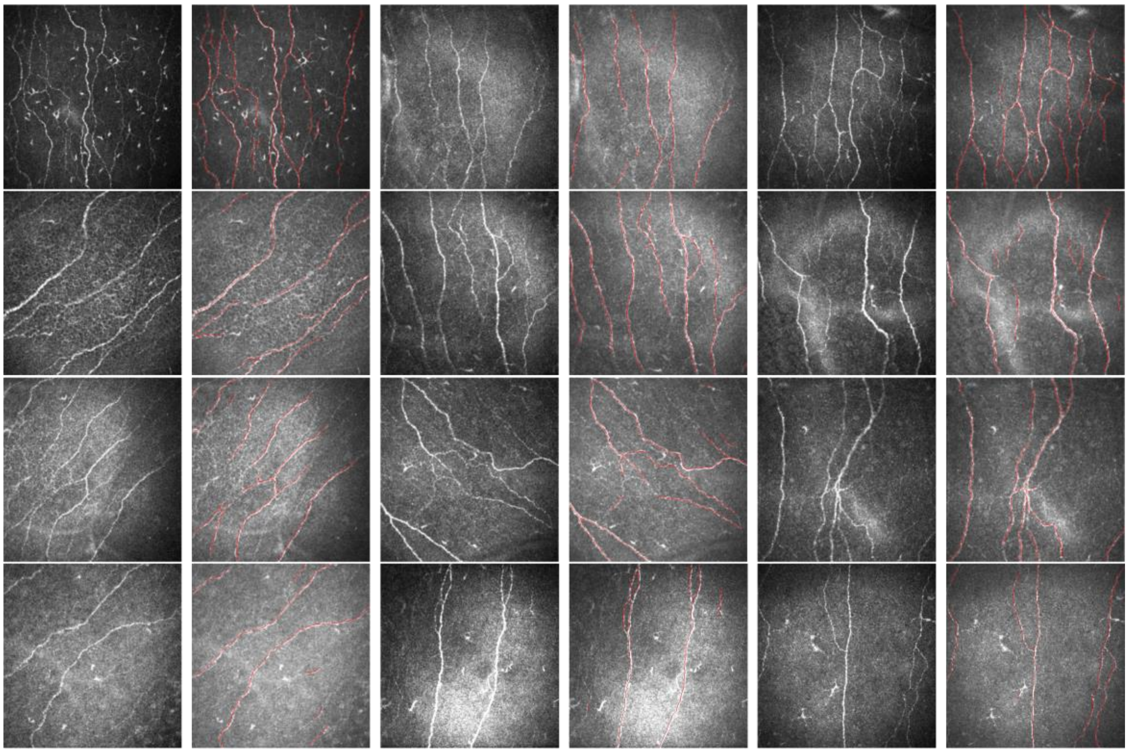

Figure SM3: Original and segmentation results from our LDLA overlaid (red) on 10 randomly selected images from Dataset 3.
